# Resilience and spirituality mediate anxiety and life satisfaction in chronically Ill older adults

**DOI:** 10.1186/s40359-023-01279-z

**Published:** 2023-08-31

**Authors:** Mohammadamin Shabani, Zahra Taheri-Kharameh, Abedin Saghafipour, Hoda Ahmari-Tehran, Sadegh Yoosefee, Mohammadali Amini-Tehrani

**Affiliations:** 1 Students Research Committee, Qom University of Medical Sciences, Qom, Iran; 2 Spiritual Health Research Center, School of Health and Religion, Qom University of Medical Sciences, Qom, Iran; 3 Department of Public Health, School of Health, Qom University of Medical Sciences, Qom, Iran; 4 Neuroscience Research Center, School of Medicine, Qom University of Medical Sciences, Qom, Iran; 5 School of Psychological Science, University of Western Australia, Perth, WA Australia; 6 Health Psychology and Behavior Medicine Research Group, Student Scientific Research Center, Tehran University of Medical Sciences, Tehran, Iran

**Keywords:** Life satisfaction, Resilience, Spirituality, Anxiety, Older people

## Abstract

**Background:**

Spirituality and psychological resilience can be considered as a protective factor for coping with anxiety in geriatric populations. The aim of the study was to investigate the structural model related to the mediating role of spirituality and psychological resilience in predicting the relationship between anxiety and life satisfaction in older adults with chronic illness.

**Methods:**

In a cross-sectional study, one hundred patients over sixty years of age from one university hospital were selected by convenience sampling. Data were collected using the Spiritual Health Scale, the Anxiety Module of the Hospital Anxiety and Depression Scale, the Connor-Davidson Resilience Scale, the Life Satisfaction Scale, and a sociodemographic questionnaire. Data analysis was performed using Partial Least Squares (PLS) structure modeling.

**Results:**

There was a positive and significant relationship between resilience, spirituality and life satisfaction of the samples. The structural model showed that spirituality, and psychological resilience mediated in the relationship between anxiety and life satisfaction directly and in indirectly in the older people with chronic disease, explaining approximately 34% of the variance in life satisfaction.

**Conclusions:**

The findings suggest that spirituality and psychological resilience can help older adults with chronic illness to negate the impact of anxiety on satisfaction, with the effect of spirituality being stronger than resilience in this relationship.

## Introduction

In recent decades, there has been notable advancement in lowering deaths and enhancing global life span. Still, this epidemiological transition has resulted in the proliferation of non-lethal illnesses, repercussions, and challenges, culminating in a global aging population [1]. In addition, with the increasing life expectancy, there is a simultaneous increase in chronic diseases and disabilities, so that there is at least one chronic disease in 84.1% of the older adults [2]. Chronic and debilitating diseases may impose various psychological issues, resulting in a high incidence of mental disorders following physical illnesses [3]. Given the increased mental disorders and the importance of aging issues, relieving anxiety and problems of this population group can be considered as a social necessity. One of the areas of concern for aging researchers is the satisfaction of the older adults with life. Life satisfaction or perceived life quality is a complex construct, which conveys the individual’s positive attitude to the world he lives in and is constituted by the pleasure feeling which the person spends much time to achieve [4].

Various factors may help preserve life satisfaction in older adults. One of the possible factors affecting life satisfaction is psychological resilience against stressors. Psychological resilience refers to an individual's capacity to effectively adjust to challenging circumstances, enabling them to withstand the pressures of daily life and to acclimate to alterations in their environment. This quality allows individuals to better manage the negative impacts of stressful events and to eventually return to their prior emotional state [5]. While psychological resilience is generally viewed as a personal characteristic that may be influenced by inherent traits and past experiences, it is also acknowledged that certain skills can be developed to promote psychological resilience in people. Therefore, psychological resilience is not an immutable personality trait but rather a malleable quality that can be cultivated through intentional effort [6]. In addition, Spirituality is a significant contributor to an individual's overall sense of contentment with life [7]. It is considered to be a valuable coping mechanism for managing stress and enhancing productivity [8]. Spiritual health encompasses a range of factors, including a person's comprehension of their purpose in life, their values, their capacity for transcendence, and their ability to connect with themselves, others, nature, and a higher power [9].

Many studies have documented significant associations between spirituality and different aspects of health. A study conducted in Iran revealed that social support and spiritual health, mediated through hope and resilience, were significant predictors of the quality of life among older adults [10]. Another study established significant correlations between Transpersonal spirituality, resiliency, and social support. Using a structural model, the researchers identified three variables that predicted spirituality: physical symptoms as the primary cause of discomfort, resiliency, and social support [11]. In line with another study, the dimension of significant meaningfulness serves as a mediator between spirituality and life satisfaction over time, suggesting that spirituality can influence how adults perceive and experience life [12]. Furthermore, another study supported the positive effects of psychological resilience on older adults' life satisfaction and emphasized the significance of family and community contexts in their resilience and life satisfaction [13].

This is due to the correlation between spirituality, psychological resilience, and higher life satisfaction in various populations. However, it's important to explore if these factors have a similar impact on older Iranians coping with chronic illnesses. Moreover, anxiety is a major concern for older adults, especially those with chronic illnesses. Investigating the protective effects of spirituality and psychological resilience in this context can help us understand how these factors mitigate anxiety's negative impact on life satisfaction. This knowledge can be utilized to develop interventions targeting these protective factors, ultimately improving the quality of life for older Iranians with chronic illnesses. Broadening research to include diverse populations is crucial for a comprehensive understanding of life satisfaction across different socio-cultural contexts. Conversely, the interplay between the aforementioned variables in the Iranian context has not been thoroughly examined. Hence, our study aimed to examine how anxiety and life satisfaction are related in older adults with chronic diseases, while also testing the mediating role of psychological resilience and spirituality. Our formulated hypotheses are as follows:

- H1: Resilience is inversely related to anxiety and positively related to life satisfaction in chronically ill older adults.
- H2: Spirituality is inversely related to anxiety and positively related to life satisfaction in chronically ill older adults.
- H3: Resilience and spirituality mediate the relationship between anxiety and life satisfaction in chronically ill older adults.

## Methods

The cross-sectional study was conducted in Qom, Iran from May to August 2021. The study population was all older adults patients with chronic diseases having referred to one university hospital. A total of 100 patients over 60 years of age were selected by convenience sampling. To be included in the study, participants were required to have a chronic illness for a minimum of six months, were Muslim, exhibit no apparent cognitive impairments (score 6 or higher in the Persian version of the abbreviated mental test) [14], be able to communicate effectively in Persian, and give their informed consent to participate in the study. After obtaining permission from the Deputy of Research and Technology of the University and also coordinating with educational and medical centers, the examiners referred to the research environment. After introducing themselves and the research and its objectives to the head of nursing, the examiners asked them to introduce any patient who gave informed consent for participations. For the included patients, the research and its objectives were stated again. If the patient agreed to participate in the study, the questionnaire was completed by the researcher using an interviewer-administered method. Exclusion criteria for the study included incomplete questionnaire responses and patient’s non-cooperation.

### Instruments

The data collection instrument in this study was five questionnaires that was completed by interview with two researchers. The first questionnaire is related to personal characteristics, including age, sex, marital status, residence status, level of education, economic status, employment status, number of hospitalizations, duration of diagnosis, which was completed based on the patient's statements and medical file. In the second questionnaire, Connor and Davidson resilience scale was used to evaluate resilience. This scale has 25 items that are scored on a Likert scale between zero (false) to five (completely true), ranging 25-125. This scale, although it measures different dimensions of resilience, has an overall score. Validity (by factor analysis and convergent and divergent validity) and reliability (by retesting method and Cronbach's alpha) of the scale have been achieved by the test manufacturers in different groups [15]. The validity and reliability of the Persian version of the resilience scale has been confirmed by Jowkar with [16]. In the study, the internal consistency coefficient of the scale with Cronbach's alpha was obtained to be 0.90 at a good level.

In the third questionnaire, the Spiritual Health Scale (FACIT-sp), there is a special questionnaire to assess spirituality in patients with chronic diseases. This standard tool has 12 items and three subscales to measure spiritual health and has been designed by Brady et al. [17]. Each sub-scale of the questionnaire has 4 phrases in that 4 questions (2, 3, 5 and 8) measure meaning, 4 phrases (1, 4, 6 and 7) peace, and 4 other phrases (9, 10, 11 and 12) faith. The participants responded to each item on a five-point Likert scale. Phrases 4 and 8 are scored inversely. The range of scores of each of the subscales varies between 0-16 and the whole scale of 0-48, and a higher score indicates better spiritual health. Considering that FACIT-Sp is a specific questionnaire for assessing spirituality in patients with chronic diseases and specific tools are more responsive to important health changes, the use of this questionnaire is recommended. It is worth mentioning that the validity and reliability of this questionnaire was confirmed by Jafari et al. in Tehran in 2013 [18]. In this study, the internal consistency coefficient of the scale was measured using Cronbach's alpha, and the obtained value of 0.83 indicated a good level of reliability.

In the fourth questionnaire, the Hospital Anxiety and Depression Scale (HADS) is a 14-item self-report questionnaire designed to assess the presence and severity of anxiety and depression symptoms in patients [19]. It has a seven-component depression subscale and a seven-part anxiety subscale. In summary, this scale is a reliable tool for assessing anxiety and depression in patients. Each test component is scored on a scale of zero to three. Therefore, the depression and anxiety subscale scores of the HADS questionnaire range from 0 to 21. The validity and reliability of this questionnaire has been confirmed by Montazeri et al. in 2008 [20].

Finally, life satisfaction was assessed using the widely used life satisfaction scale designed by Diner et al. (1985) [21]. This scale has the 5-choice five items designed to measure an individual's overall judgment of life satisfaction, which is theoretically based on a comparison of living conditions with predetermined standards. Because different people may have different ideas about the composition of a good life, this scale is designed to measure people's overall life satisfaction. This scale was normalized to be used in Iran by Maroufizadeh et al [22]. The scale's internal consistency coefficient was assessed using Cronbach's alpha, and the resulting value was 0.93, indicating a highly reliable measure.

### Data analysis

Study data were analyzed using descriptive statistics, Pearson correlation coefficient and variance-based structural equation modeling. Significance level in all tests was considered less than 0.05. To perform structural equation modeling, the partial least squares method using PLS-3 software application was employed. To evaluate the structural model, the coefficient of determination (R^2^), β values, redundancy validity (CV-red), common validity index (CV-com), goodness-of-fit index (GOF) and average variance extracted index (AVE) were used [23]. R^2^ index determines the proportion of variance in the dependent variable that can be explained by the independent variables, while β values the strength of an effect from a single independent variable on the outcome. The CV-red index indicates the structural quality of the model and the CV-com index indicates the common validity of each latent construct. Positive values of these indices for all variables indicate the appropriate quality of the model. AVE values are recommended by Fornell and Larcker (1981), and a preferred value of 0.50 and above means that the measurement model explains more than 50% of the variance of the latent construct. To evaluate the overall fit of the structural model, the goodness-of-fit index (GOF) index and R^2^ index have been used. According to Tennhaus et al. (2004) 0.1, 0.25 and 0.36 were introduced as weak, medium and strong values, respectively, to confirm the model fit [24]. Our independent variable was anxiety, while wellbeing, life satisfaction was the dependent variable and resilience was the expected mediating variable. Mediation analysis was carried out using Bootstrapping method to estimate 95% confidence interval of the direct effect (denoted by c’) of the independent variable (i.e., anxiety) on the outcome (i.e., life satisfaction), and indirect effects (denoted by a × b) of each proposed mediators.

## Results

### Sample characteristics

The study sample consisted of one hundred older adults with chronic diseases with a mean age of 62.66 ± 7.92 years and in the age range of 60-87 years. Of them, 51% participants were women, 71% married, 49% housewives, 44% illiterate and 40% had insufficient income. The highest prevalence of underlying diseases among the studied participants was heart disease (69%). Table 1 presents demographic features of the participants.

**Table 1:** Demographic characteristics of the study sample (*n* = 100)

Factor	**Number**
**Gender**	
Male	49
Female	51
**Marital status**	
Single	2
Married	71
Divorced	3
Widow	24
**Job Status**	
Employed	30
Unemployed	21
Housewife	49
**Level of Education**	
Illiterate	44
Elementary school	28
Middle school	5
High school	4
Diploma	10
Academic degree	9
**Income status**	
Insufficient	40
Low	24
Middle	30
High	6
**Chronic disease**	
Cardiovascular	69
High Blood Pressure	5
Diabetes	16
Kidney Failure	10
Other	7
**Smoking Status**	
Non smoker	66
Smoking cessation	17
Smoker	17
Age (years) Mean(SD)	62.66 (7.92)
Disease duration Mean(SD)	8.58 (8.02)

### Correlational results

There was a positive and significant relationship between resilience, spirituality and life satisfaction of the older adults (*P* <0.01). However, there was a negative and significant relationship between anxiety and other study variables (*P* <0.05) (Table 2).

**Table 2:** Descriptive statistics of study variables in elderly with chronic diseas

Variables	**Average**	**SD**	**Cronbach's alpha**	**Life Satisfaction**	**Anxiety**	**Spirituality**
Life Satisfaction	14.62	7.58	0.868	1		
Anxiety	1.82	0.28	0.774	-0.223^*^	1	
Spirituality	27.20	6.90	0.787	0.699^**^	-0.489^**^	1
Resilience	85.30	21.50	0.958	0.632^**^	-0.297^**^	0.761^**^

^**^P < 0.01

^*^P < 0.05

### The Structural model

The quality indicators of the structural model are shown in Table 3. The coefficient of determination of life satisfaction was 0.336, showing that anxiety, resilience and spirituality could predict 34% of life satisfaction changes in the elderly with chronic diseases, which is at an intermediate level. In addition, the AVE values were greater than 0.50, which indicate the convergent validity of all constructs. The GoF index was calculated as 0.549, which is strong according to the set criteria. Thus, the overall model fit was confirmed.

**Table 3:** Structural quality indicators of life satisfaction model in elderly with chronic disease

Variables	R-Square	CV-red	CV-com	AVE	CR
Life Satisfaction	0.336	0.167	0.435	0.577	0.8
Resilience	0.459	0.333	0.633	0.78	0.946
Spirituality	0.878	0.312	0.401	0.707	0.878
Anxiety	_	_	0.611	0.798	0.941

Accordingly, the structural model indicated that the relationship between anxiety and life satisfaction was completely mediated by spirituality and resilience Table 4.

**Table 4:** Path analysis of life satisfaction model (*n* = 100)

Variables	Path confidence	T	*P*-value	95% CI
Resilience—> Life Satisfaction	0.286	2.429	0.015	0.06, 0.51
Spirituality—> Life Satisfaction	0.388	3.116	0.002	0.14,0 .62
Anxiety—> Life Satisfaction	-0.075	0.564	0.573	-0.34, 0.16
Anxiety—> Resilience	-0.677	-10.963	0.000	-0.78, -0.54
Anxiety—> Spirituality	-0.686	-12.837	0.000	-0.77, -0.57
Anxiety—> Spirituality—> Life Satisfaction	-0.266	-2.789	0.005	-0.45, -0.09
Anxiety—> Resilience—> Life Satisfaction	-0.194	-2.390	0.017	-0.35,-0.04
Total indirect effect	-0.460	-4.975	0.000	-0.63,-0.27

**Fig. 1:**
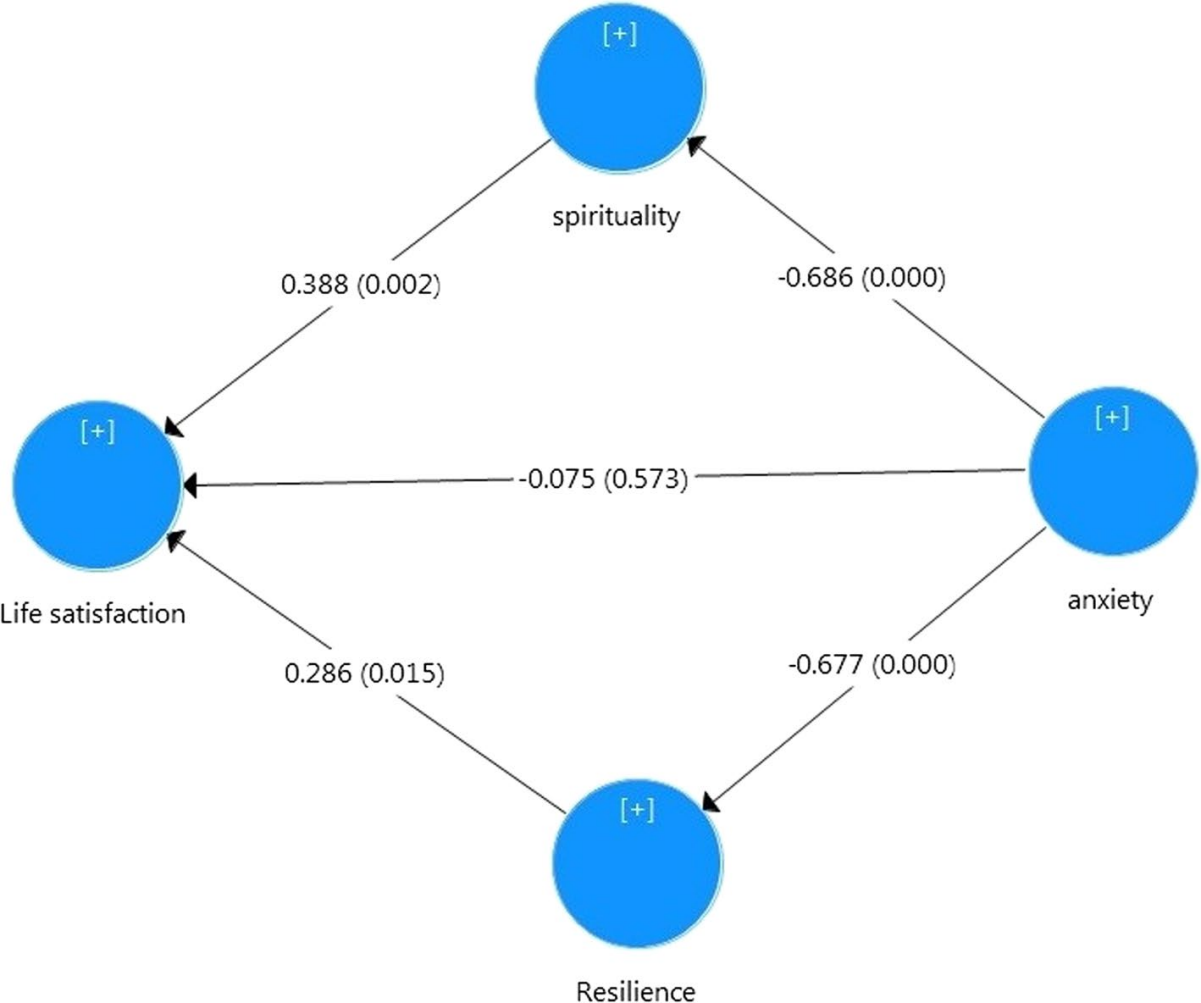
Structural model of study variables

In that, the direct effect of anxiety on life satisfaction was reduced to non-significant and near to zero value (c’ = 0.08, *P* = 0.573, 95% confidence interval = [-0.34, 0.16]). However, there were negative indirect effects through spirituality (a1 × b1 = -0.27, *P* = 0.005, 95% confidence interval = [-0.45, -0.09]) and resilience (a2 × b2 = -0.19, *P* = 0.017, 95% confidence interval = [-0.35, -0.04]). Figure 1 shows the structural model of life satisfaction in standardized estimations.

## Discussion

The study sought to predict life satisfaction pattern in older adults with chronic disease based on anxiety, with resilience and spirituality components acting as mediators. The findings support the hypothesis that resilience and spirituality mediate the link between anxiety and life satisfaction in chronically ill older adults. In other words, anxiety can negatively affect the overall life satisfaction of older individuals dealing with chronic disease. However, the study also highlights how spirituality and psychological resilience can buffer the adverse effects of anxiety on life satisfaction, serving as protective factors. This underscores the importance of addressing not only the physical health concerns but also the mental and spiritual well-being of older adults with chronic disease. Consistent with our study, Kwok (2020) demonstrated that in order to improve the health-related quality of life in patients with chronic diseases like Parkinson's, it is necessary to strengthen spirituality in addition to alleviating physical and mental symptoms. This integrated approach enables emotional stability, acceptance, and a positive mindset toward both positive and negative health experiences throughout the illness [25]. According to Munawar's (2018) findings, there is a significant relationship between life satisfaction, spiritual intelligence, and religiosity in Muslim older adults. Life satisfaction is negatively associated with awareness, grace, meaning, truth, and excellence, which are components of spiritual intelligence. Conversely, life satisfaction is positively related to religion. Gender differences and attendance of religious services also impact life satisfaction and spiritual intelligence in Muslim older adults [26]. Moons (2019) revealed that religiosity and the importance of religion and spirituality positively correlate with quality of life, life satisfaction, and health behaviors. However, in secular countries, religion and spirituality exhibit a negative association with physical and mental health. Religiosity and spirituality are independent predictors across various professions, with differing effects in different countries [27]. A study conducted in Denmark emphasized the lack of a common understanding of spirituality within a modern secular environment, leading to confusion and misunderstandings when discussing spiritual beliefs and practices [28]. The study also found that religious participants hold distinct moral values not found among secular participants. This suggests that a sense of community and shared moral values within a religious congregation contribute to a higher quality of life. It raises questions about potential benefits of secular communities that promote similar shared values and a sense of belonging, as well as the need for further research on the moral values promoted by religious congregations and their contribution to overall well-being [29].

Furthermore, the study identified resilience as a mediator in the relationship between stress and life satisfaction, aligning with other studies [30–32]. Jones (2019) found a positive association between resilience, increased positive impact, and life satisfaction. Higher levels of spirituality and resilience significantly contributed to positive psychological outcomes for individuals with spinal cord injury and their family members, both independently and in combination [31]. Resilience acts as a protective factor against the negative impacts of stress on life satisfaction. Resilient individuals possess psychological resources such as optimism, composure, and openness, which help them find positive meaning in challenging situations. These characteristics contribute to higher levels of life satisfaction by mitigating the negative effects of stress [33]. Since resilience skills can be acquired, teaching these skills may improve individuals' ability to handle stress, thereby enhancing their satisfaction with work and life. However, there is a lack of relevant research on interventions for older adults, demonstrating the need for further investigation in this area.

Several limitations should be acknowledged in this study. The use of non-random sampling and a small sample size can significantly limit the generalizability of the findings. Additionally, the study's focus on older adults hospitalized in a teaching hospital and the non-compliance of certain patients in completing the questionnaires can be regarded as constraints in the present study.

The implications of these findings for clinical practice are significant. Healthcare providers who work with chronically ill elders may consider incorporating spirituality and resilience-building interventions into their care plans to help improve psychological well-being and quality of life. This may involve providing access to spiritual counseling, mindfulness-based interventions, or other forms of psychosocial support. Additionally, it is important for healthcare providers to recognize the potential impact of chronic illness on mental health and to screen for symptoms of anxiety and other psychological distress. It is worth noting, the relationships between resilience, spirituality, anxiety, and life satisfaction in chronically ill elders are complex and multifaceted. The findings from this study suggest that resilience and spirituality may play important roles in promoting psychological well-being and enhancing quality of life in this population. Further research is needed to better understand the mechanisms underlying these relationships and to develop effective interventions to improve the psychological well-being of chronically ill elders.

## Conclusion

Findings showed that resilience and spirituality have a positive and significant direct and indirect effect on life satisfaction in the older adults with chronic diseases. This highlights the importance of addressing spirituality in healthcare for patients with chronic illness. Healthcare providers should consider incorporating spiritual practices and beliefs into treatment plans to improve overall well-being and life satisfaction.

## Data Availability

The datasets used and/or analysed during the current study available from the corresponding author on reasonable request.

## Abbreviations

HADS: Hospital Anxiety and Depression Scale
CV-red: Redundancy validity
CV-com: Common validity index
AVE: Average variance extracted index
GOF: Goodness-of-fit index
HRQOL: Health-related quality of life
